# Revealing common differential mRNAs, signaling pathways, and immune cells in blood, glomeruli, and tubulointerstitium of lupus nephritis patients based on transcriptomic data

**DOI:** 10.1080/0886022X.2023.2215344

**Published:** 2023-06-19

**Authors:** Haifang Zhao, Dongxia Zheng

**Affiliations:** Department of Nephrology, Dongying People’s Hospital, Dongying, China

**Keywords:** Lupus nephritis, differentially expressed mRNAs, LTF, MX1, cell cycle, immune cell infiltration

## Abstract

Lupus nephritis (LN) is a potentially fatal autoimmune disease. The purpose of this study was to find potential key molecular markers of LN to aid in the early diagnosis and management of the disease. Datasets GSE99967_blood, GSE32591_glomeruli, and GSE32591_tubulointerstitium were included in this study. Differentially expressed mRNAs (DEmRNAs) were identified between the normal control and LN groups using the limma package in R. Common DEmRNAs in the three datasets were taken. Subsequently, functional enrichment analysis, immune correlation analysis, receiver operating characteristic (ROC) curve analysis and real-time polymerase chain reaction (RT-PCR) verification were performed. In this study, 11 common DEmRNAs were obtained and all of them were up-regulated. In protein–protein interaction (PPI) networks, we found that MX dynamin like GTPase 1 (MX1) and radical S-adenosyl methionine domain containing 2 (RSAD2) had the highest interaction score (0.997). Functional enrichment analysis revealed that MX1 and RSAD2 were enriched in influenza A and hepatitis C signaling pathways. The area under the curve (AUC) values of interferon-induced protein 44 (IFI44) and MX1 in GSE32591_glomeruli and GSE32591_tubulointerstitium datasets are 1, which is worthy of further study on their diagnostic value and molecular mechanism. The xCell analysis showed abnormal distribution of granulocyte-macrophage progenitor (GMP) cells in blood, glomeruli, and tubulointerstitium. Pearson’s correlation analysis found that GMP cells were significantly correlated with lactotransferrin (LTF) and cell cycle. Identification of common DEmRNAs and key pathways in the blood, glomeruli, and tubulointerstitium of patients with LN provides potential research directions for exploring the molecular mechanisms of the disease.

## Introduction

Lupus nephritis (LN) is a complication of systemic lupus erythematosus (SLE) involving the kidneys. Most SLE patients develop LN within 5 years of SLE diagnosis [1]. LN is a common clinical complication associated with SLE mortality. It is also an important cause of death in SLE patients [2]. Renal biopsy is the gold standard for diagnosis of LN. The clinical manifestations of LN are usually subtle, and the common manifestations are proteinuria, microscopic hematuria, and renal tubular abnormalities [3]. Treatment strategies for LN aim to achieve rapid or at least partial remission to prevent flares and preserve renal function, reducing morbidity, and mortality [4,5]. The choice of treatment modality is largely determined by histological type, activity, and chronic indicators, including immunosuppressive agents, adjuvants, and symptomatic drugs [6]. Although the treatment plan is constantly optimized with the further research, the treatment of LN is still not satisfactory due to the complex and diverse etiology of LN.

Single-cell transcriptomic studies of kidneys from LN patients and normal control samples revealed the complexity of the LN kidney immune population [7]. B cell abnormality plays a key role in the pathogenesis and immunotherapy of LN [8]. In mouse models of LN, T-cell-specific silencing of signal transducer and activator of transcription 3 (STAT3) blocks their ability to help B cells produce autoantibodies and induce cell tissue infiltration [9]. Extrarenal factors in the pathogenesis of LN include complex combinations of genetic variants that differ in each patient, which explains the variability in clinical presentation. SLE occurs when genetic variation disrupts those mechanisms that normally ensure immune tolerance to nuclear self-antigens. Moreover, renal internal factors are also involved in the mechanism of autoimmune regulation [10]. Therefore, exploring the distribution of immune cells in patients with LN is helpful for the study of its pathogenesis and immunotherapy.

LN is a potentially fatal autoimmune disease with complex molecular regulatory mechanisms. The T cell receptor (TCR) repertoire of LN patients and healthy controls was assessed by high-throughput sequencing and significant differences in TCR diversity were found between the two groups [11]. Urinary forkhead box P3 (FOXP3) mRNA is elevated in patients with active LN and is associated with SLEDAI and disease severity [12]. Interleukin-34 (IL-34) is highly expressed in human mesangial cells of LN patients and is negatively regulated by the Wnt pathway [13]. The WNT/β-catenin signaling pathway is activated in patients with LN [14]. Although LN has a large number of molecular studies, its specific molecular mechanism is still unclear. In addition, genes often have different expression patterns in different tissues. In order to diagnose and manage patients more directly and easily, it is necessary to identify common molecular mechanisms in different tissues. Therefore, in order to understand the underlying molecular mechanisms of LN and explore new biomarkers to aid in the early management and treatment of LN, we performed bioinformatic analysis.

In this study, mRNA expression data were downloaded from Gene Expression Omnibus (GEO). According to false discovery rate (FDR) < 0.05 and |log2fold change| (|log2FC|) > 1, 11 differentially expressed mRNAs (DEmRNAs) were obtained with universal differential expression in blood samples, glomerular samples, and tubulointerstitial compartment samples. Meanwhile, the xCell tool was used to analyze the distribution of immune cells in LN patients and normal controls. Subsequently, Pearson’s correlation analysis was used to calculate the correlation between DEmRNAs, differential signaling pathways, and differential immune cells. Finally, cell cycle signaling pathway and lactotransferrin (LTF) were significantly correlated with granulocyte-macrophage progenitor (GMP) cells. Identification of common DEmRNAs, immune cells and key pathways in the blood, glomeruli and tubulointerstitium of patients with LN provides potential clinical research directions for exploring the molecular mechanisms of the disease. Moreover, the identification of novel biomarkers may contribute to early clinical diagnosis, management, and treatment.

## Materials and methods

### Datasets collecting

To acquire the gene expression profile data of LN, we searched the widely used GEO database. Datasets that meet the following criteria will be included in our study: (1) the dataset must contain genome-wide mRNA transcriptome expression data; (2) data from LN patients and control samples (blood, tissue); (3) this study considers both standardized and raw datasets. After filtering, GSE99967 (including blood samples from 29 patients with active LN and 17 controls) and GSE32591 (including glomerular samples from 32 patients with LN and 14 pre-transplant healthy living donors (GSE32591_glomeruli), and tubulointerstitial compartment samples from 32 patients with LN and 15 pre-transplant healthy living donors (GSE32591_tubulointerstitium)) were selected for subsequent analyses. Scale standardization was carried out for 10,993 common mRNAs among three datasets (Figure S1A). Then, the combat function of the R language SVA package was used for batch correction of samples (Figure S1B).

### Differential expression analysis

The limma package in R was used to identify DEmRNAs in three datasets (GSE99967_blood, GSE32591_glomeruli, and GSE32591_tubulointerstitium). FDR < 0.05 and |log2FC| > 1 were the screening criteria for DEmRNAs. With R package ‘pheatmap’, hierarchical clustering analysis was performed. Subsequently, common DEmRNAs in the three datasets were taken. In order to explore the interaction relationship between common DEmRNAs, the String database (https://string-db.org/) was used to construct the protein–protein interaction (PPI) network of common DEmRNAs. Gene Ontology (GO) and Kyoto Encyclopedia of Genes and Genomes (KEGG) pathway enrichment analysis was performed on DEmRNAs and common DEmRNAs using GeneCodis 4.0 (*p* value_adj < 0.05) [15–17].

### Gene set variation analysis (GSVA)

The GSVA package was used for GSVA analysis of expression matrix of all genes. The ‘c2.cp.kegg.v7.4.symbols.gmt’ was selected as the reference gene set, and the gene set enrichment score of a single sample was obtained. Subsequently, differential signaling pathways with consistent directions in the three datasets were screened.

### Diagnostic analysis

To assess the potential diagnostic value of common DEmRNAs, we performed diagnostic analyses in GSE99967_blood, GSE32591_glomeruli and GSE32591_tubulointerstitium data. The pROC package in R was used for diagnostic analysis. Diagnostic ability of common DEmRNAs was evaluated by the area under the curve (AUC) values in the receiver operating characteristic (ROC) curve. The larger the AUC, the higher the diagnostic accuracy [18]. The sensitivity and specificity at the cutoffs were determined referring to previous report [19].

### Immune correlation analysis

The xCell tool [20] (https://xcell.ucsf.edu/) was used to calculate the distribution of immune cells in each sample, and xCell score of 64 immune cells in all samples was obtained. The xCell score was collated into an immune cell infiltration matrix, and the types of immune cells that differed between the two groups were obtained by difference analysis. Subsequently, Pearson’s correlation analysis was used to calculate the correlation between the common differential immune cells in the three datasets and common DEmRNAs in the GSE99967_blood dataset. In addition, Pearson’s correlation analysis was used to calculate the correlation between common differential immune cells and common signaling pathways in the GSE99967_blood dataset.

### Real-time polymerase chain reaction (RT-PCR) validation

Blood samples from LN and healthy individuals were collected for RT-PCR validation. The inclusion criteria for LN were as follows: (1) patients diagnosed with LN, that is, those with renal involvement on the basis of being diagnosed with SLE (meeting the diagnostic criteria of ACR or ERA-EDTA); (2) patients diagnosed with LN by rheumatologists or nephrologists. The exclusion criteria for LN were as follows: (1) SLE patients with serious damage to other organs besides kidney involvement; (2) patients with end-stage renal disease; (3) patients taking immunosuppressants for a long time (or those taking immunosuppressants for several consecutive days at the time of inclusion); (4) patients with other severe diseases, such as tumors; (5) patients during pregnancy; (6) patients with renal involvement due to hypertension/diabetes. The inclusion criteria for the control group were healthy individuals without immune system disease or other diseases. Ten human whole blood samples were included in this study, including five LN blood samples and five normal control blood samples (Table S1). Total RNA was extracted from blood samples using TRIzol kit for RT-PCR verification. Then, FastKing cDNA first-strand synthesis kit was used for mRNA reverse transcription. If the synthesized cDNA needs to be stored for a long time, it should be stored at −20 °C or lower. SuperReal PreMix Plus (SYBR Green) was used for amplification. RT-PCR was performed by ABI StepOne Plus fluorescence quantitative PCR instrument. Glyceraldehyde-3-phosphate dehydrogenase (GAPDH) and actin beta (ACTB) were used as internal reference genes. 2^–ΔΔCt^ method was used for relative quantitative analysis of data [21]. The 2^–ΔΔCt^ represents the change factor of target gene expression in the disease group compared with the control group. –ΔΔCt >0 and –ΔΔCt <0 represent up-regulated and down-regulated, respectively.

### ROC analysis and expression validation based on GSE81622 and GSE112943 datasets

The GSE81622 dataset includes peripheral blood mononuclear cells (PBMC) samples from 15 patients with LN and 25 normal controls. The GSE112943 dataset includes kidney samples from 14 patients with LN and seven normal controls. To further validate the potential value of common DEmRNAs, ROC analysis, and expression validation were performed based on the GSE81622 and GSE112943 datasets.

### Statistical analysis

R software (version 4.0.5) was used to perform all statistical analyses. The limma package in R was used to identify DEmRNAs. FDR < 0.05 and |log2FC| > 1 were the screening criteria for DEmRNAs. The pROC package in R was used for diagnostic analysis. Wilcoxon’s method was used to analyze the statistical difference of xCell score between LN and normal control groups. *p* < .05 was considered as statistical significance. Pearson’s correlation analysis was used to calculate correlations between common differential immune cells and common DEmRNAs and common signaling pathways in the GSE99967_blood data.

## Results

### Identification of DEmRNAs

**Figure 1.**
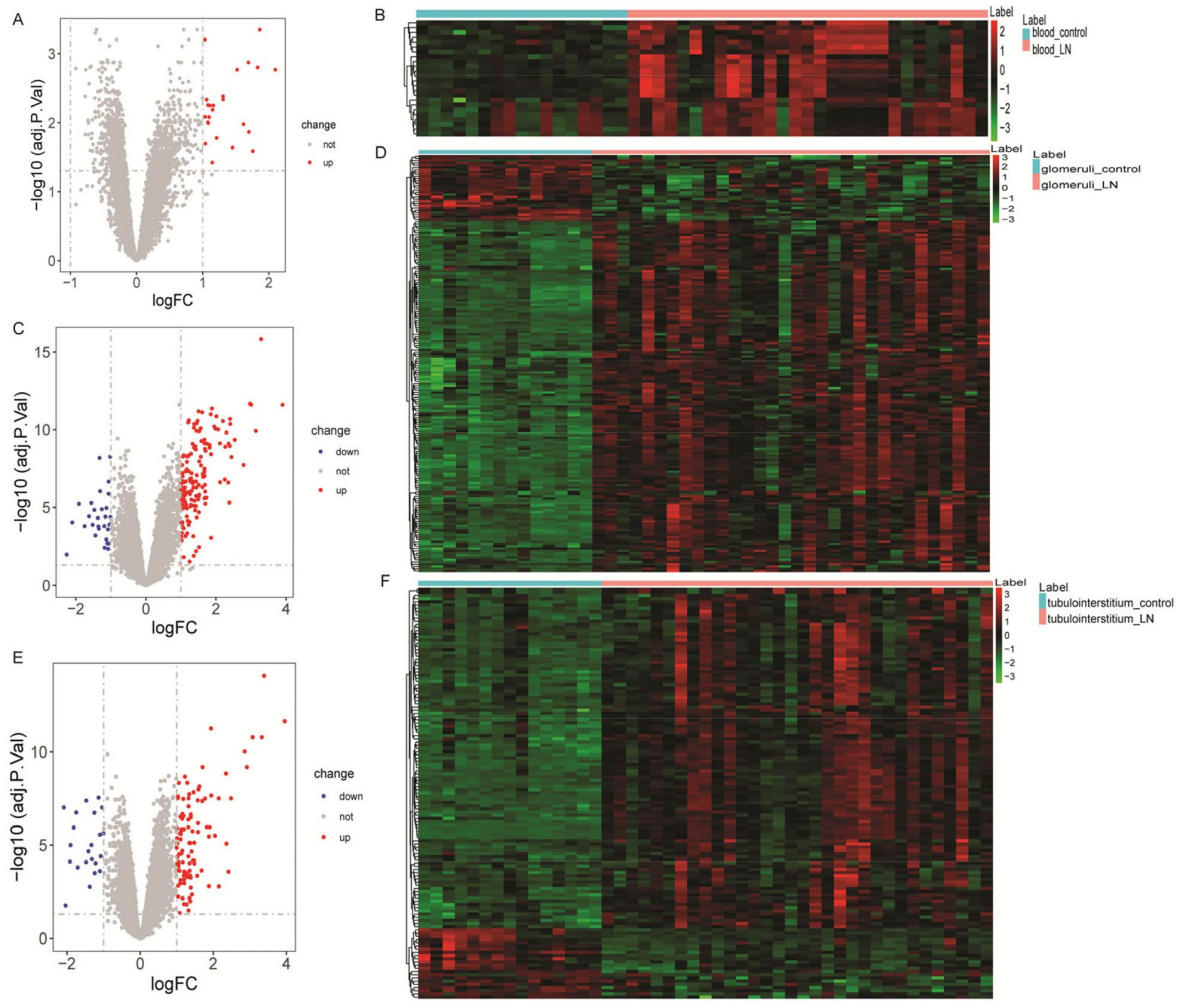
Volcano plot and hierarchical clustering analysis of DEmRNAs between LN and controls. (A) Volcano map of DEmRNAs in the GSE99967_blood dataset. The red and blue dots represent the up-regulated and down-regulated of mRNAs in LN blood samples, respectively. (B) Heat map of hierarchical cluster analysis of DEmRNAs in the GSE99967_blood dataset. Row and column represented DEmRNAs and samples, respectively. The color scale represented the expression levels. (C) Volcano map of DEmRNAs in the GSE32591_glomeruli dataset. The red and blue dots represent the up-regulated and down-regulated of mRNAs in LN glomerular samples, respectively. (D) Heat map of hierarchical cluster analysis of DEmRNAs in the GSE32591_glomeruli dataset; (E) volcano map of DEmRNAs in the GSE32591_tubulointerstitium dataset. The red and blue dots represent the up-regulated and down-regulated of mRNAs in LN tubulointerstitial compartment samples, respectively. (F) Heat map of hierarchical cluster analysis of DEmRNAs in the GSE32591_tubulointerstitium dataset.

**Figure 2.**
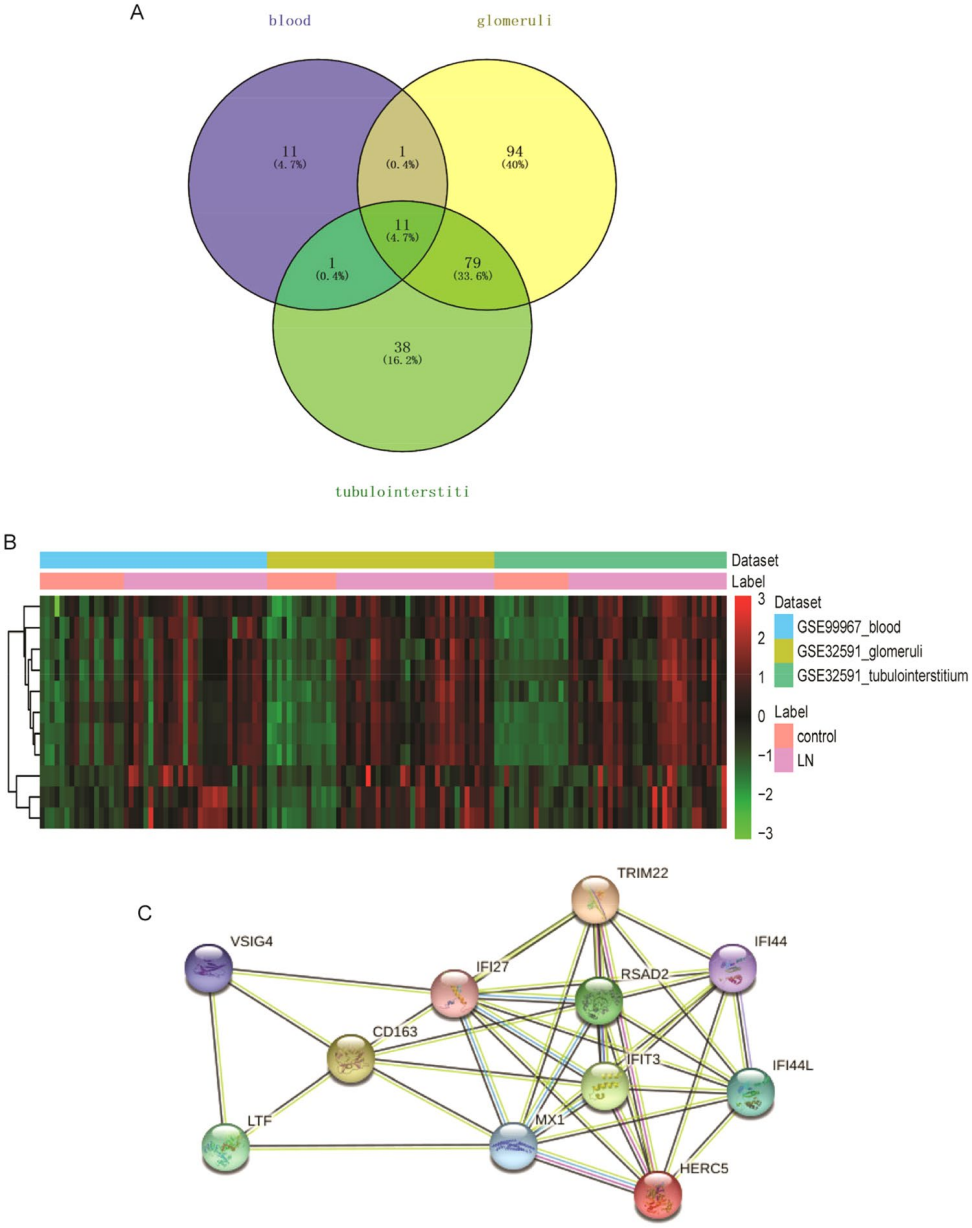
Identification of common DEmRNAs. (A) Venn diagram for three datasets; (B) hierarchical clustering analysis of common DEmRNAs in three datasets. Row and column represented DEmRNAs and samples, respectively. The color scale represented the expression levels. (C) Protein–protein interaction (PPI) among common DEmRNAs.

According to FDR < 0.05 and |log2FC| > 1, 24, 185, and 129 DEmRNAs were identified from the GSE99967_blood (24 DEmRNAs were up-regulated), GSE32591_glomeruli (156 DEmRNAs were up-regulated and 29 DEmRNAs down-regulated), and GSE32591_tubulointerstitium (107 DEmRNAs were up-regulated and 22 DEmRNAs down-regulated) datasets. The volcano map and heat map of DEmRNAs are shown in Figure 1. Then, GO and KEGG functional enrichment analysis of DEmRNAs in GSE99967_blood, GSE32591_glomeruli, and GSE32591_tubulointerstitium datasets were performed. The results showed that DEmRNAs were significantly enriched in numerous immune and inflammation-related biological processes (BPs) (Figures S2–S4). Subsequently, common DEmRNAs in the three datasets were taken. A total of 11 common DEmRNAs were obtained, namely tripartite motif containing 22 (TRIM22), CD163 molecule (CD163), LTF, V-set and immunoglobulin domain containing 4 (VSIG4), radical S-adenosyl methionine domain containing 2 (RSAD2), interferon alpha inducible protein 27 (IFI27), HECT and RLD domain containing E3 ubiquitin protein ligase 5 (HERC5), interferon-induced protein with tetratricopeptide repeats 3 (IFIT3), interferon-induced protein 44 (IFI44), interferon-induced protein 44 like (IFI44L), and MX dynamin like GTPase 1 (MX1), and their expressions were all up-regulated (Figure 2(A,B)). There are a total of 37 interaction pairs in the PPI network, among which MX1and RSAD2 have the highest score (0.997) (Figure 2(C)).

### Functional enrichment analysis of common DEmRNAs

**Figure 3.**
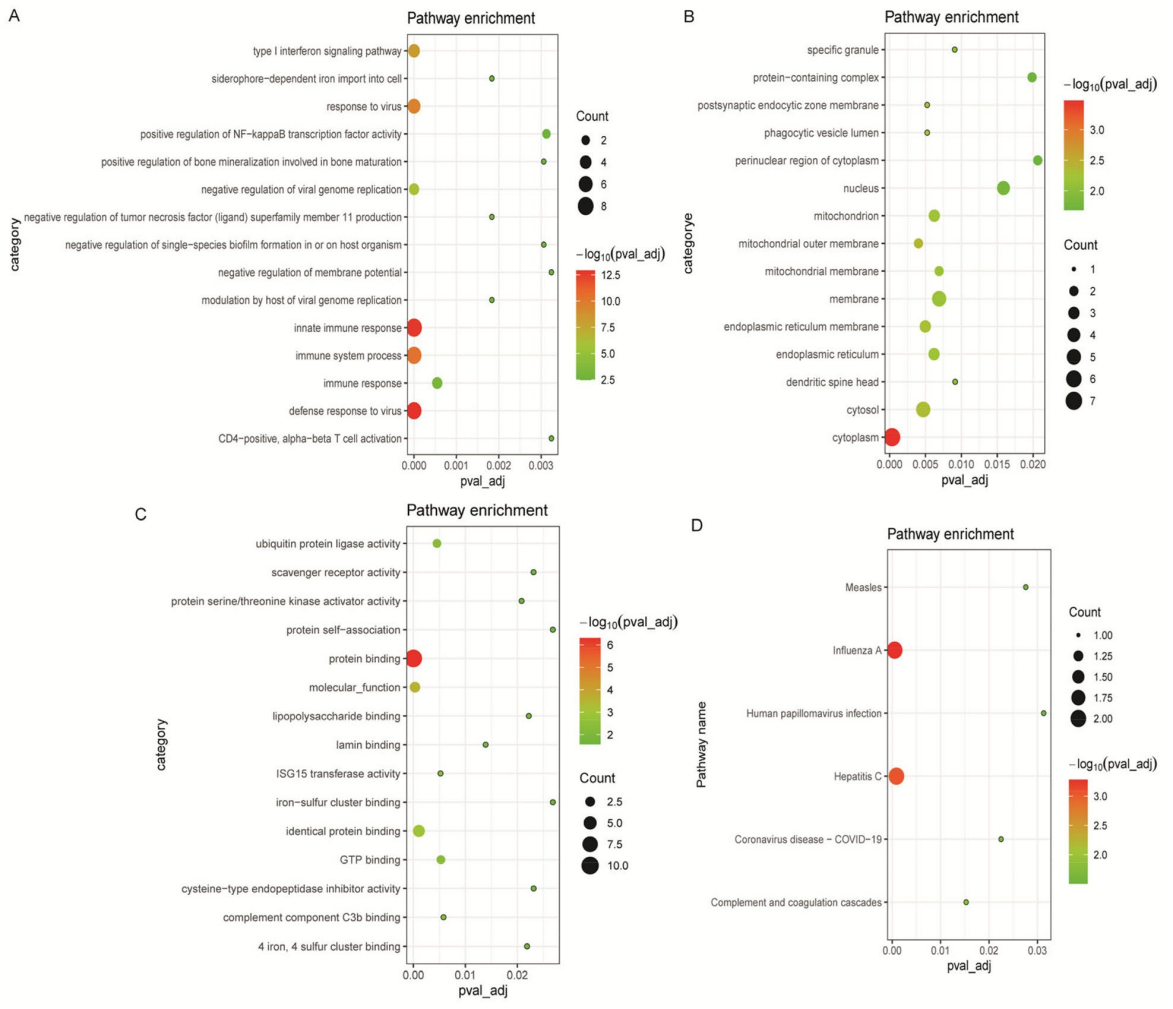
Significantly enriched GO terms and KEGG pathways of common DEmRNAs. (A) Top 15 biological process (BP) terms in GO functional enrichment; (B) top 15 cellular component (CC) terms in GO functional enrichment; (C) top 15 molecular function (MF) terms in GO functional enrichment; (D) KEGG functional enrichment analysis. The *x*-axis shows *p* value_adj of DEmRNAs enriched in GO terms or KEGG pathways and the *y*-axis shows GO terms or KEGG pathways. The color scale represented –lg *p* value_adj.

In order to better understand the BPs involved in common DEmRNAs, GO and KEGG functional enrichment analyses were performed for common DEmRNAs. GO functional analysis found that common DEmRNAs in BP term were mainly enriched in immune-related BPs such as innate immune response, immune system process, defense response to virus and type I interferon (IFN) signaling pathway (Figure 3(A)). Common DEmRNAs found in cell composition (CC) were mainly distributed in cytoplasm, cytosol, and membrane (Figure 3(B)). Common DEmRNAs found in molecular function (MF) term were mainly involved in protein binding, identical protein binding, and molecular_function (Figure 3(C)). In addition, KEGG functional enrichment analysis revealed that common DEmRNAs were mainly enriched in influenza A, hepatitis C, complement and coagulation cascades and other biological signaling pathways (Figure 3(D)).

### Diagnostic analysis of common DEmRNAs

To evaluate the potential diagnostic value of TRIM22, CD163, LTF, VSIG4, RSAD2, IFI27, HERC5, IFIT3, IFI44, IFI44L and MX1, ROC analysis was performed. AUC value between 0.5 and 0.6, diagnostic accuracy is ‘bad’, AUC value between 0.6 and 0.7, diagnostic accuracy is ‘sufficient’, AUC value between 0.7 and 0.8, diagnostic accuracy is ‘good’, AUC value between 0.8 and 0.9, diagnostic accuracy is ‘very good’, and the AUC value is between 0.9 and 1.0, the diagnostic accuracy is ‘excellent’ [19]. The results showed that the AUC values of all common DEmRNAs were greater than 0.6 (Figure S5–S7), which suggested that they may be potential diagnostic molecular markers. It is worth noting that the AUC values of IFI44 and MX1 in GSE32591_glomeruli and GSE32591_tubulointerstitium datasets are 1, which is worthy of further study on their diagnostic value and molecular mechanism.

### GSVA

GSVA analysis found that there were 12 common differential signaling pathways in the three datasets, of which four pathways (leukocyte transendothelial migration, cell cycle, O-glycan biosynthesis, and cytosolic DNA sensing pathway) had the same direction and were all up-regulated (Table S2). This suggests that leukocyte transendothelial migration, cell cycle, O-glycan biosynthesis, and cytosolic DNA sensing pathway are important in LN.

### Immune correlation analysis

**Figure 4.**
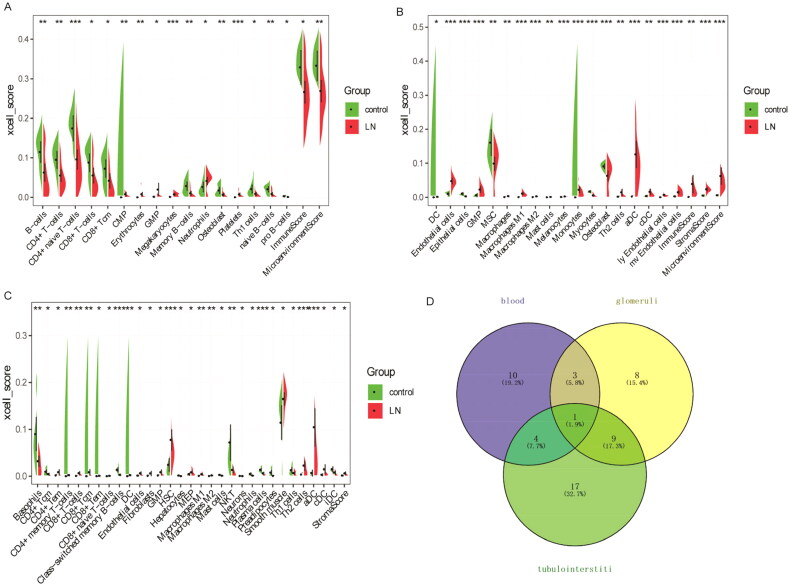
xCell scores of immune cell types between LN and controls. (A) xCell scores of 18 immune cell types between LN and controls in the GSE99967_blood dataset; (B) xCell scores of 21 immune cell types between LN and controls in the GSE32591_glomeruli dataset; (C) xCell scores of 31 immune cell types between LN and controls in the GSE32591_tubulointerstitium dataset. (D) Venn diagram shows that GMP is a common differential immune cell type in the three datasets. *p < .05, **p < .01, and ***p < .001.

**Figure 5.**
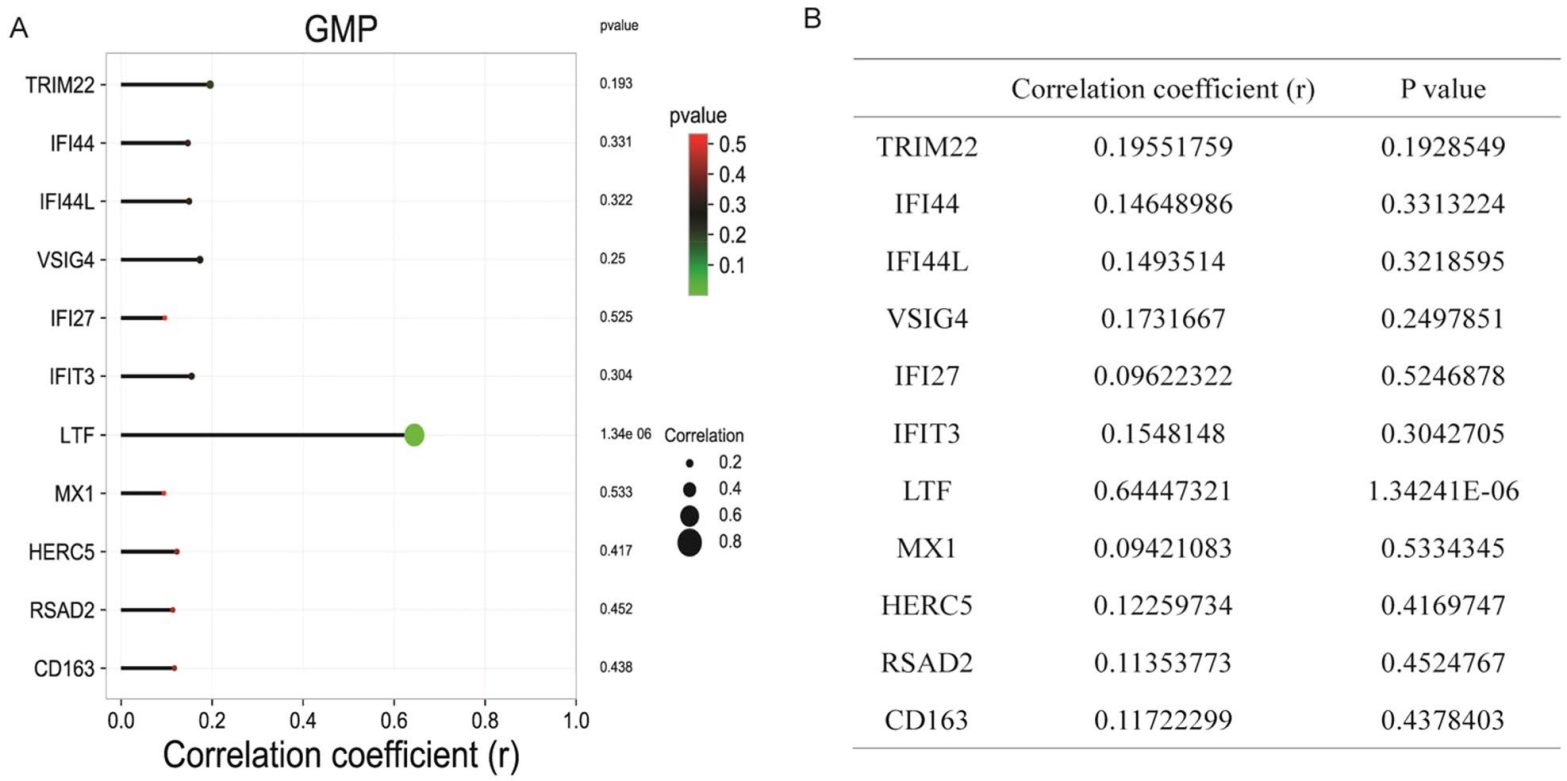
Correlation between the expression levels of common DEmRNAs and the immune infiltration level of GMP cell. (A) Bar diagram of correlation between the expression levels of common DEmRNAs and the immune infiltration level of GMP cell. The horizontal axis represents the correlation coefficient, and the color of the points at the end of the line segment represents the *p* value. (B) Correlation coefficients between the expression levels of common DEmRNAs and the immune infiltration level of GMP cell.

**Figure 6.**
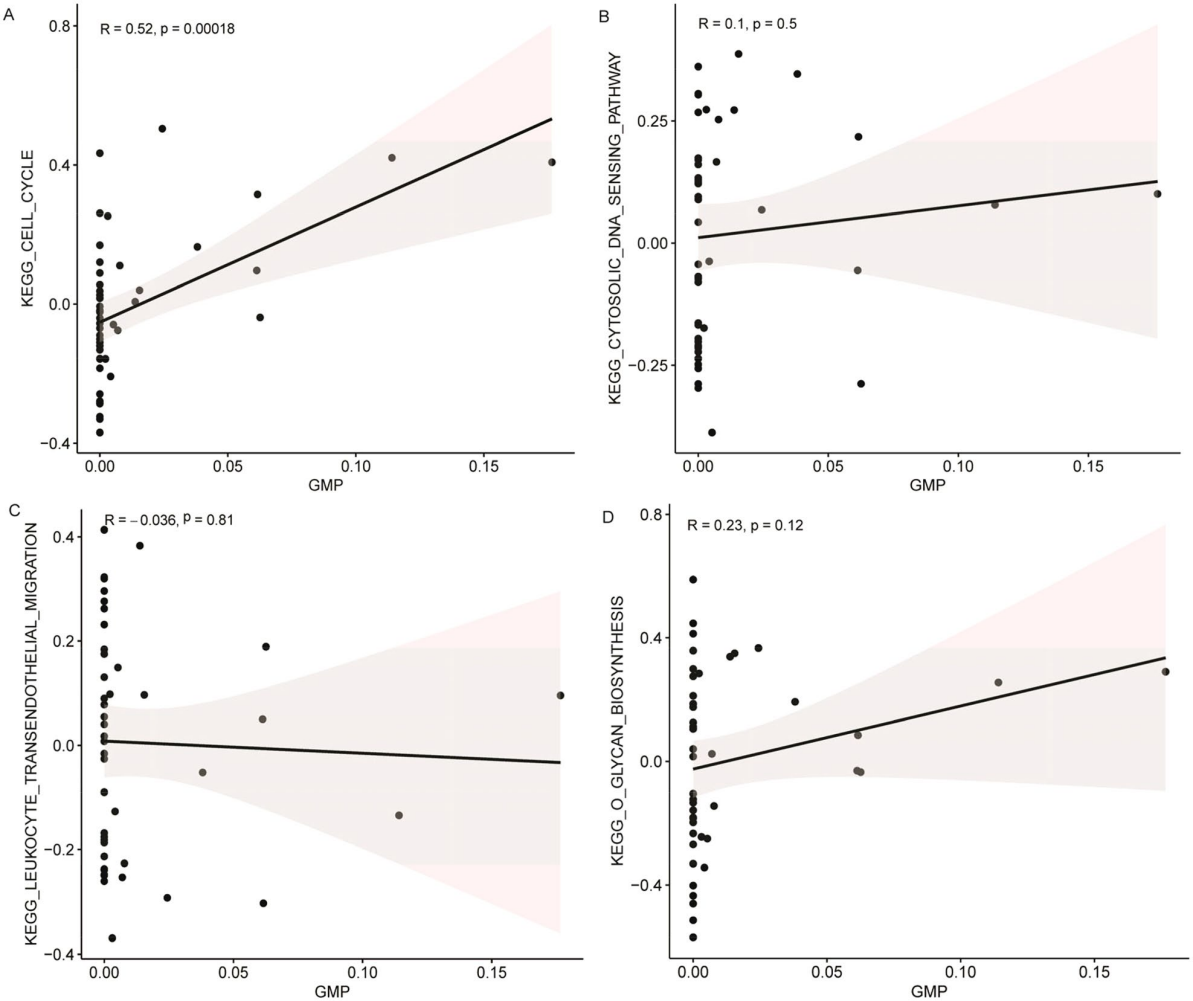
Correlation between the activity of common differential signaling pathways and the immune infiltration level of GMP cell based on GSE99967_blood dataset. (A) Correlation between cell cycle signaling pathway and GMP cell; (B) correlation between cytosolic DNA sensing pathway and GMP cell; (C) correlation between leukocyte transendothelial migration signaling pathway and GMP cell; (D) correlation between o glycan biosynthesis signaling pathway and GMP cell. 0 < *R* < 1 represents positive linear correlation, –1 < *R* < 0 represents negative linear correlation, and *R* = 0 represents no linear correlation. *p* < .05 represents statistical significance, *p* > .05 represents no statistical significance.

Through differential analysis, 18, 21, and 31 immune cell types were significantly different in the GSE99967_blood, GSE32591_glomeruli, and GSE32591_tubulointerstitium datasets, respectively (Figure 4(A–C) and Table S3). Among them, GMP is the common differential immune cell type in the three datasets (Figure 4(D)). Pearson’s correlation analysis found that LTF was significantly correlated with GMP immune cells (correlation coefficient (*r*) = 0.644473, *p* value = 1.34241E–06) (Figure 5). These results suggest that LTF up-regulation in LN may mediate the immunomodulatory effect of GMP cell. Subsequently, the correlation between GMP and four common differential signaling pathways was analyzed. Pearson’s correlation analysis showed that GMP cell was significantly correlated with cell cycle signaling pathway (correlation coefficient (*r*) = 0.52, *p* value = 0.00018) (Figure 6). This further indicates that the cell cycle signaling pathway plays an important role in the progression of LN. In Figure 6, each dot represents a sample. The *y*-axis is the GSVA score. The *x*-axis is the score of GMP cell immune infiltration calculated by the xCell tool.

### RT-PCR validation

**Figure 7.**
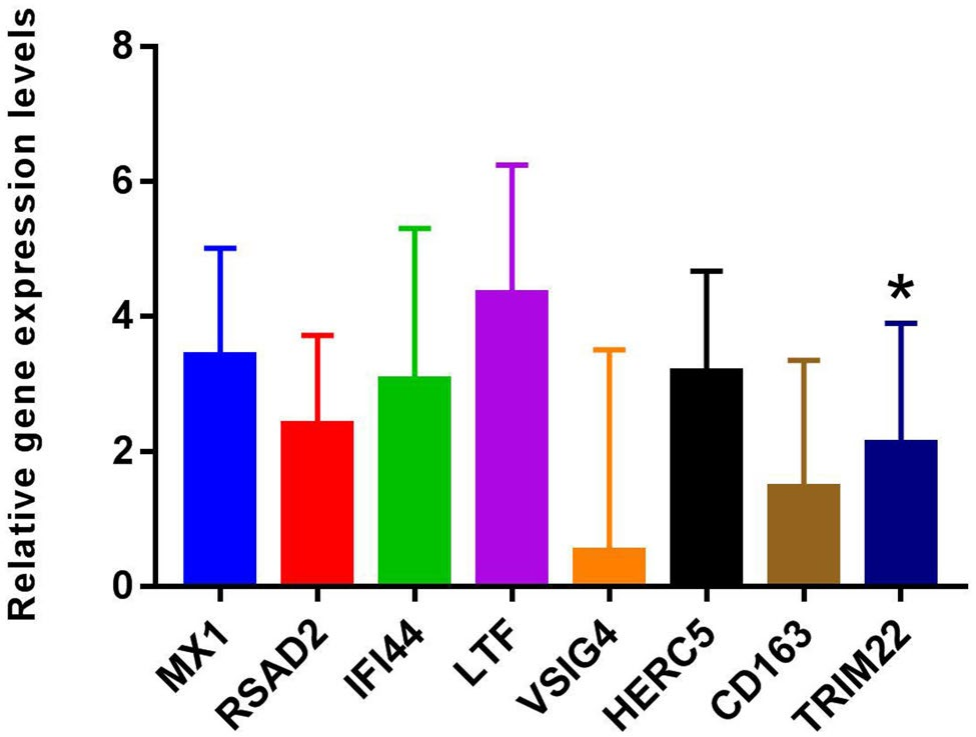
The expressions of MX1, RSAD2, IFI44, LTF, VSIG4, HERC5, CD163, and TRIM22 were verified by RT-PCR based on five LN blood samples and five normal control blood samples. 2^–ΔΔCt^ method was used for relative quantitative analysis of data. The 2^–ΔΔCt^ represents the change factor of target gene expression in the disease group compared with the control group. –ΔΔCt >0 and –ΔΔCt <0 represent up-regulated and down-regulated, respectively. *p < .05.

MX1, RSAD2, IFI44, LTF, VSIG4, HERC5, CD163, and TRIM22 were selected for RT-PCR validation. Primers of MX1, RSAD2, IFI44, LTF, VSIG4, HERC5, CD163, and TRIM22 are shown in Table S4. RT-PCR results showed that the expression levels of MX1, RSAD2, IFI44, LTF, VSIG4, HERC5, CD163, and TRIM22 were up-regulated trend in LN compared with the control (Figure 7 and Table S5). Among them, the differential expression of TRIM22 was significant. DEmRNAs expression trends verified by RT-PCR were consistent with bioinformatic analysis. However, most of the DEmRNAs lack significance in RT-PCR validation, which may be caused by the small sample size. Therefore, it is necessary to expand the sample for further research.

### ROC analysis and expression validation of common DEmRNAs in GSE81622 and GSE112943 datasets

To further validate the potential value of common DEmRNAs, ROC analysis and expression validation were performed based on the GSE81622 and GSE112943 datasets. The results of GSE81622 dataset showed that common DEmRNAs were up-regulated in the LN group compared with the control group (Figure S8). In addition, expression validation of common DEmRNAs was also performed in the GSE112943 dataset. Except for VSIG4, other common DEmRNAs were also up-regulated in the LN group (Figure S8). The ROC analysis results based on the GSE81622 and GSE112943 datasets showed that the AUC values of all common DEmRNAs were greater than 0.6 (Figures S10 and S11), which again suggested that they may be potential diagnostic molecular markers.

## Discussion

Based on differential expression screening criteria, common DEmRNAs (TRIM22, CD163, LTF, VSIG4, RSAD2, IFI27, HERC5, IFIT3, IFI44, IFI44L, and MX1) in the three datasets were screened. MX1, formerly known as myxovirus (influenza) resistance 1, is a potential marker of LN activity in the kidney [22]. MX1 is highly expressed in kidney tissue of LN patients before immunosuppressive treatment, but decreased after immunosuppressive treatment [17]. In addition, MX1 expression in LN mesangial cells is associated with antiviral innate immunity [23]. A study showed that TRIM22, IFI27, IFIT3, IFI44, IFI44L, MX1, and RSAD2 are hub genes of LN, and are positively correlated with monocyte infiltration in the glomeruli and M2 macrophage infiltration in the tubulointerstitium [24]. IFI27, IFI44, IFI44L, and RSAD2 may also play a role in SLE by interfering with the type I IFNs pathway [25,26]. Furthermore, a genome-wide DNA methylation study found hypomethylation of IFIT3, MX1, IFI44L, and TRIM22 in naive CD4+ T cells of SLE patients [27]. However, the specific mechanisms of them in LN remain unclear. In this study, MX1 and RSAD2 had the highest interaction scores (0.997) in PPI networks. This implies that MX1 and RSAD2 may play a role in the progression of LN through mutual influence. Moreover, KEGG analysis showed that MX1 and RSAD2 were enriched in influenza A and hepatitis C signaling pathways. Concurrent hepatitis C virus infection in patients with LN is associated with decreased survival [28]. Patients with SLE experienced remission of nephrotic syndrome after treatment of chronic hepatitis C virus infection [29]. Influenza is an important risk factor for LN and SLE [30,31]. These results further imply that MX1 and RSAD2 may play molecular regulatory role in LN disease progression by co-regulating influenza A and hepatitis C. Notably, the AUC value of MX1 and IFI44 in GSE32591_glomeruli and GSE32591_tubulointerstitium datasets is 1, which is worth further exploring its clinical diagnostic value.

Urinary soluble CD163 may be a biomarker of renal disease activity in LN, and its levels vary with LN activity and duration of treatment [32,33]. HERC5 has abnormal methylation modification in LN and is an important regulatory molecule in the pathogenesis of LN [34,35]. In this study, CD163 and HERC5 were common DEmRNAs, and ROC analysis found that they have potential diagnostic value. This lays a theoretical foundation for future research. VSIG4 (also known as CRIg, Z39IG) is specifically expressed in macrophages and is involved in the clearance of immune complexes and autologous cells, its important in regulating innate and adaptive immune responses [36,37]. Moreover, VSIG4 plays an important role in the treatment of LN [38,39]. As a specific complement receptor of macrophages, VSIG4 was enriched in complement and coagulation cascades pathway in this experiment. Therefore, we hypothesized that VSIG4 may play a role in LN by participating in the regulation of the complement and coagulation cascades pathway.

LN is a potentially fatal autoimmune disease, and the distribution and activation state of immune cells plays an important regulatory role in its disease progression [7,40]. In the present study, xCell analysis found a large number of abnormal immune cell infiltration in blood, glomeruli, and tubulointerstitium compared with normal controls. Among them, GMP is the common differential immune cell type in GSE99967_blood, GSE32591_glomeruli, and GSE32591_tubulointerstitium datasets. GMP cells have the potential to differentiate into granulocytes and macrophages and play a crucial role in the immune system [41,42]. Pearson’s correlation analysis found that GMP cells were significantly correlated with LTF (*r* = 0.644473, *p* value = 1.34241E–06) and cell cycle signaling pathway (*r* = 0.52, *p* value = .00018) in this study. One study showed that the expression of LTF was up-regulated in LN [43], which was consistent with our results. Furthermore, the expression of LTF in the B cells of female SLE patients was significantly lower than that of male SLE patients, and this difference was influenced by estrogen [44]. Therefore, we speculate that the abnormal expression of LTF may affect the distribution of GMP cells in the LN immune microenvironment and the role of immune regulation. In addition, the abnormality of GMP cells may affect the cell cycle of LN-associated cells, thereby affecting the progression of LN. The correlation of GMP cells with LTF and cell cycle provides a potential research direction for further study of the molecular mechanism of LN.

However, this study has certain limitations. First, all the analysis data of this study were downloaded from GEO public database, lacking clinical sample verification. Therefore, a large number of clinical samples need to be collected for further research. Second, the specific molecular mechanisms of the key mRNAs and signaling pathways identified in LN are unclear. Therefore, extensive *in vitro* studies are needed to further explore the specific molecular mechanisms of LN.

## Conclusions

In this study, 11 common DEmRNAs in blood, glomeruli, and tubulointerstitium were obtained. In PPI networks, we found that MX1 and RSAD2 had the highest interaction score. Moreover, MX1 and RSAD2 were enriched in influenza A and hepatitis C signaling pathways. These results imply that MX1 and RSAD2 play an important role in LN disease progression. In addition, the AUC values of IFI44 and MX1 in GSE32591_glomeruli and GSE32591_tubulointerstitium datasets are 1, which is worthy of further study on their diagnostic value and molecular mechanism. The xCell analysis found a large number of abnormal immune cell infiltration in blood, glomeruli, and tubulointerstitium compared with normal controls. Remarkably, Pearson’s correlation analysis found that GMP cells were significantly correlated with LTF and cell cycle. These results indicate that LTF and cell cycle play a role in immune regulation of LN. In short, the identification of common DEmRNAs and key pathways in the blood, glomeruli, and tubulointerstitium of patients with LN provides potential research directions for exploring the molecular mechanisms of the disease. In addition, the identification of potential diagnostic markers contributes to the early diagnosis and management of LN.

## Supplementary Material

Supplemental Material

Supplemental Material

Supplemental Material

Supplemental Material

Supplemental Material

Supplemental Material

## Data Availability

All data generated or analyzed during this study are included in this published article. We searched for LN public data from GEO (http://www.ncbi.nlm.nih.gov/geo). The accession numbers are GEO: GSE99967, GSE32591_glomeruli, and GSE32591_tubulointerstitium.
