# Supplementary material for: Revealing common differential mRNAs, signaling pathways, and immune cells in blood, glomeruli, and tubulointerstitium of lupus nephritis patients based on transcriptomic data
Source: Ren Fail. 2023 Jun 19;45(1):2215344. doi: 10.1080/0886022X.2023.2215344 (PMC10281411; doi:10.1080/0886022X.2023.2215344)
Supplement: Supplemental Material [file IRNF_A_2215344_SM1960.pdf]

Table S5 RT-PCR data

-△△Ct	MX1	RSAD2	IFI44	LTF	VSIG4	HERC5	CD163	TRIM22
No. 1	2.00835427	2.46185022	-0.04113215	1.41043353	-2.78667433	2.31023349	0.25983075	-0.91668239
No. 2	6.15118974	4.21474098	4.18063386	5.20713292	0.37392301	5.60551254	3.78282687	3.27604043
No. 3	3.10833931	0.55620604	1.91662572	6.35162290	0.61811583	2.61918193	1.79221745	3.34864181
No. 4	3.11078312	2.00328016	3.25280023	3.55984738	-0.88944949	3.53021987	2.48081912	2.01597781
No. 5	2.68234477	2.74200757	5.91472824	5.13749156	5.28321626	1.74557908	-1.03200542	2.87153986
Mean value	3.412	2.396	3.045	4.333	0.520	3.162	1.457	2.119
SD	1.5960	1.3201	2.2564	1.9122	2.9850	1.5110	1.8840	1.7779

-ΔΔCt >0 and -ΔΔCt <0 represent up-regulated and down-regulated, respectively.

Information Classification: General
