# Supplementary material for: Revealing common differential mRNAs, signaling pathways, and immune cells in blood, glomeruli, and tubulointerstitium of lupus nephritis patients based on transcriptomic data
Source: Ren Fail. 2023 Jun 19;45(1):2215344. doi: 10.1080/0886022X.2023.2215344 (PMC10281411; doi:10.1080/0886022X.2023.2215344)
Supplement: Supplemental Material [file IRNF_A_2215344_SM1930.pdf]

Table S4 Primers used for RT-PCR

Primer name	Primer sequence (5' to 3')
GAPDH-F (internal reference)	5-GGAGCGAGATCCCTCCAAAAT-3
GAPDH-R (internal reference)	5-GGCTGTTGTCATACTTCTCATGG-3
ACTB-F (internal reference)	5-CATGTACGTTGCTATCCAGGC-3
ACTB-R (internal reference)	5-CTCCTTAATGTCACGCACGAT-3
MX1-F	5-GGTGGTCCCCAGTAATGTGG-3
MX1-R	5-CGTCAAGATTCCGATGGTCCT-3
RSAD2-F	5-TTGGACATTCTCGCTATCTCCT-3
RSAD2-R	5-AGTGCTTTGATCTGTTCCGTC-3
IFI44-F	5-GGTGGGCACTAATACAACTGG-3
IFI44-R	5-CACACAGAATAAACGGCAGGTA-3
LTF-F	5-CCCAGGAACCGTACTTCAGC-3
LTF-R	5-GTGCCACAACGGCATGAGA-3
VSIG4-F	5-CTGCTGGACACAGTTCCTCTG-3
VSIG4-R	5-GGGCTATCCAGGAAGAGAGGT-3
HERC5-F	5-AACTGGGAGAGCCTTGTGGT-3
HERC5-R	5-CTGGCATATGACGGCTGTTT-3
CD163-F	5-TTTGTCAACTTGAGTCCCTTCAC-3
CD163-R	5-TCCCGCTACACTTGTTTTCAC-3
TRIM22-F	5-CTGTCCTGTGTGTCAGACCAG-3
TRIM22-R	5-TGTGGGCTCATCTTGACCTCT-3

Information Classification: General
