# Supplementary material for: Revealing common differential mRNAs, signaling pathways, and immune cells in blood, glomeruli, and tubulointerstitium of lupus nephritis patients based on transcriptomic data
Source: Ren Fail. 2023 Jun 19;45(1):2215344. doi: 10.1080/0886022X.2023.2215344 (PMC10281411; doi:10.1080/0886022X.2023.2215344)
Supplement: Supplemental Material [file IRNF_A_2215344_SM1927.pdf]

Table S3 Immune cell types with significant differences in GSE99967_blood, GSE32591_glomeruli and GSE32591_tubulointerstitium datasets

	GSE99967_blood dataset		GSE32591_glomeruli dataset		GSE32591_tubulointerstitium dataset	
	Immune cell types	P value	Immune cell types	P value	Immune cell types	P value
1	B-cells	0.00300537507662774	DC	0.0491179868876488	Basophils	0.00362088426281115
2	CD4+ T-cells	0.00290866653749011	Endothelial cells	1.43093671790411e-10	CD4+ Tcm	0.0366765513533642
3	CD4+ naive T-cells	0.00021712560511606	Epithelial cells	0.000726475256372644	CD4+ Tem	0.0282893913430459
4	CD8+ T-cells	0.0264794174297637	GMP	0.000510848924319495	CD4+ memory T-cells	0.00806822909239267
5	CD8+ Tcm	0.0297204945302985	MSC	0.00527970386323876	CD8+ T-cells	0.00348610933377873
6	CMP	0.00491707550053423	Macrophages	0.00900844903672028	CD8+ Tcm	0.00937110584249129
7	Erythrocytes	0.00253879614658486	Macrophages M1	8.69221285993816e-07	CD8+ Tem	0.0351637063108684
8	GMP	0.022891467238933	Macrophages M2	0.000572475197402538	CD8+ naive T-cells	0.0214895753367838
9	Megakaryocytes	6.86316810929375e-05	Mast cells	0. 00745946667139955	Class-switched memory B-cells	0.000229034078035471
10	Memory B-cells	0.00307334673353489	Melanocytes	5.64682745183537e-05	DC	0.00335354042523917
11	Neutrophils	0.0454204902706169	Monocytes	9.3379958577008e-06	Endothelial cells	0.0139405602890722
12	Osteoblast	0.00995020111906324	Myocytes	6.91894736152005e-08	Fibroblasts	0.0455885460331266
13	Platelets	0.000526436962174253	Osteoblast	0.00066649369828948	GMP	0.0181156542907425
14	Th1 cells	0.0275425463867425	Th2 cells	0.00978627980214566	HSC	0.000108170609960777
15	naive B-cells	0.00204106120196128	aDC	2.17408844006204e-07	Hepatocytes	0.0153216466166386
16	pro B-cells	0.0444895465136251	cDC	0.0045409305186147	MEP	0.0223695354008586
17	ImmuneScore	0.0155804027035815	ly Endothelial cells	4.36456428963566e-07	Macrophages M1	0.00106028362804639

Information Classification: General

18	MicroenvironmentScore	0.00634135845061598	mv Endothelial cells	2.39211620543043e-06	Macrophages M2	0.00241838968587531
19			ImmuneScore	0.00518324600613621	Mast cells	0.0235933221577804
20			StromaScore	1.41365104739975e-10	NKT	0.00588196650124427
21			MicroenvironmentScore	5.48659380994818e-05	Neurons	0.0393982086567183
22					Neutrophils	0.0241852895128995
23					Plasma cells	0.000299082786701105
24					Preadipocytes	0.0130334465628271
25					Smooth muscle	0.0119773056495542
26					Th1 cells	0.0416092545828784
27					Th2 cells	0.000873559602079623
28					aDC	2.43024185894263e-05
29					cDC	0.0271946194607758
30					iDC	0.0349223731500046
31					StromaScore	0.0164194726726754

Information Classification: General
