# Supplementary material for: Revealing common differential mRNAs, signaling pathways, and immune cells in blood, glomeruli, and tubulointerstitium of lupus nephritis patients based on transcriptomic data
Source: Ren Fail. 2023 Jun 19;45(1):2215344. doi: 10.1080/0886022X.2023.2215344 (PMC10281411; doi:10.1080/0886022X.2023.2215344)
Supplement: Supplemental Material [file IRNF_A_2215344_SM1902.pdf]

Table S1 Clinical information of individuals in the RT-PCR

Serial number	Group	Age	Sex	Clinical manifestations (hematuria, leukocyturia, etc.)	Proteinuria (g/24h)	Persistent proteinuria	Medication status (immunosuppressive/ glucocorticoid therapy)	Other comorbidities
1	LN group	26	Female	Hematuria, leukocyturia	3.26	Yes	No	No
2	LN group	24	Female	Hematuria	No	No	No	No
3	LN group	26	Female	Leukocyturia	0.87	Yes	No	No
4	LN group	41	Female	Hematuria	0.11	No	No	No
5	LN group	71	Female	Leukocyturia	1.15	Yes	No	No
6	Control group	35	Female					
7	Control group	56	Male					
8	Control group	59	Male					
9	Control group	49	Female					
10	Control group	59	Male					

Information Classification: General
