# Supplementary material for: Revealing common differential mRNAs, signaling pathways, and immune cells in blood, glomeruli, and tubulointerstitium of lupus nephritis patients based on transcriptomic data
Source: Ren Fail. 2023 Jun 19;45(1):2215344. doi: 10.1080/0886022X.2023.2215344 (PMC10281411; doi:10.1080/0886022X.2023.2215344)
Supplement: Supplemental Material [file IRNF_A_2215344_SM1894.pdf]

Supplementary Figure

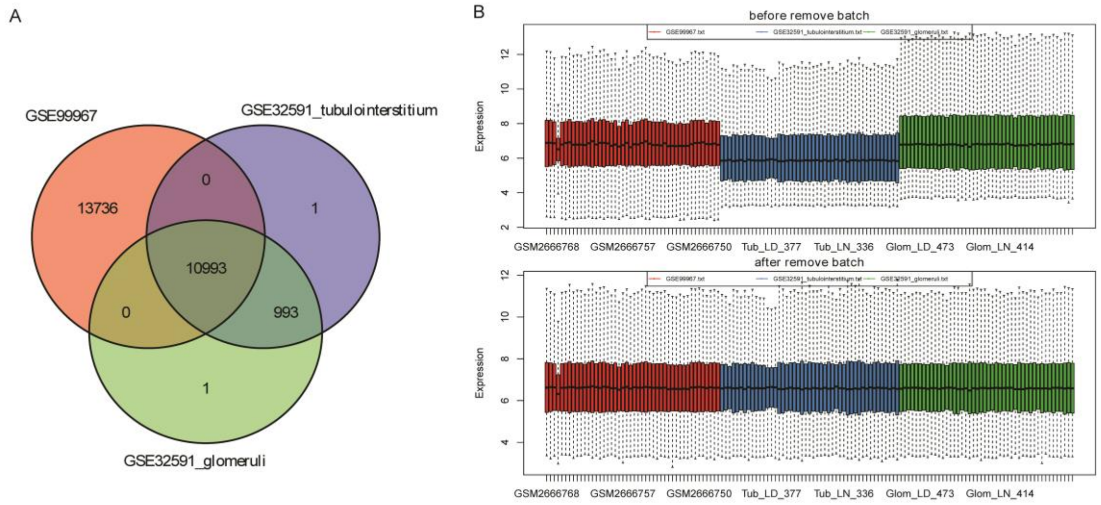

Figure S1: Distribution of mRNA data and removal of batch effects.

A: Venn diagram of mRNA intersection in GSE99967_blood, GSE32591_glomeruli and GSE32591_tubulointerstitium datasets; B: Boxplot before and after removing batch effects.

Information Classification: General

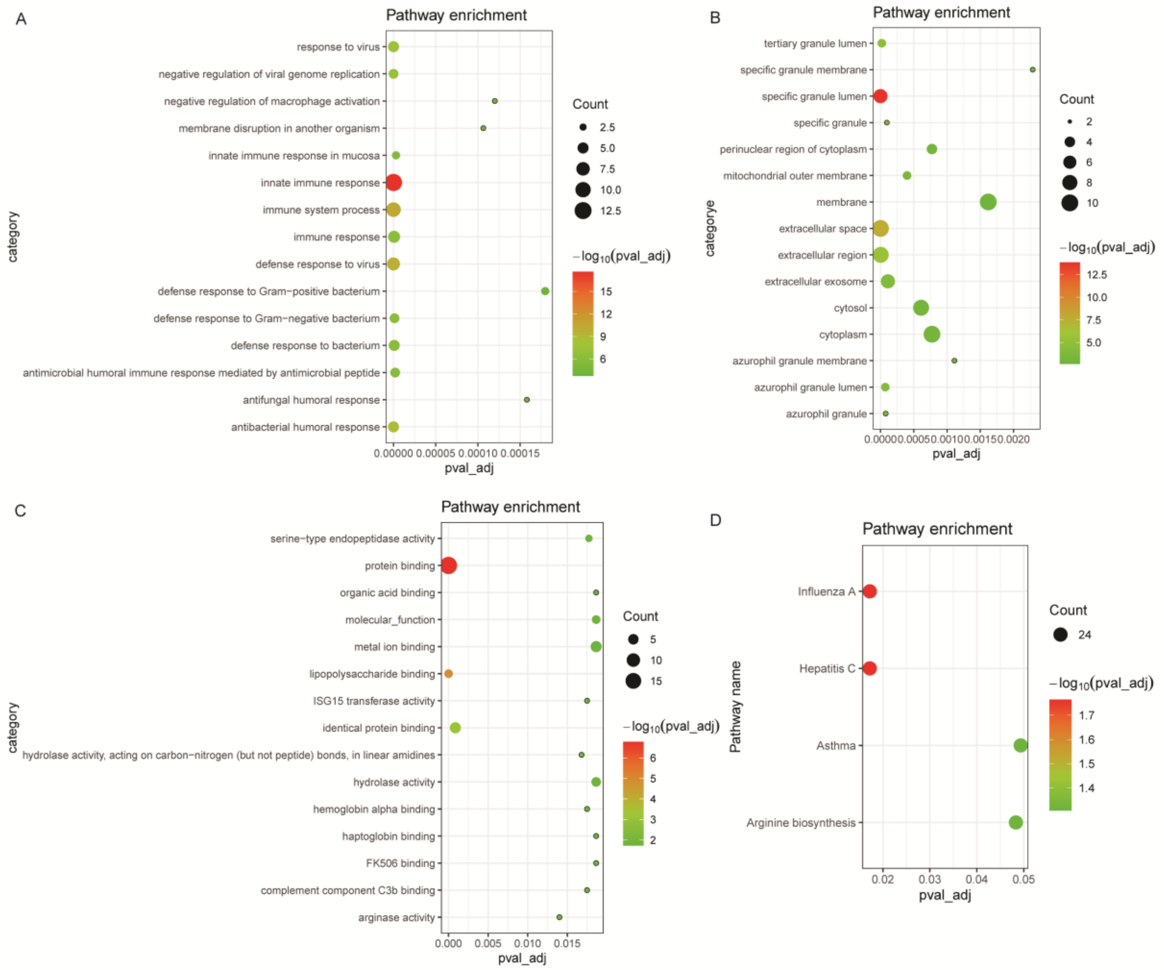

Figure S2: Significantly enriched GO terms and KEGG pathways of DEmRNAs in GSE99967_blood dataset.

A: Top 15 biological process (BP) terms in GO functional enrichment; B: Top 15 cellular component (CC) terms in GO functional enrichment; C: Top 15 molecular function (MF) terms in GO functional enrichment; D: KEGG functional enrichment analysis. The x-axis shows P-value_adj of DEmRNAs enriched in GO terms or KEGG pathways and the y-axis shows GO terms or KEGG pathways. The color scale represented -lg p-value_adj.

Information Classification: General

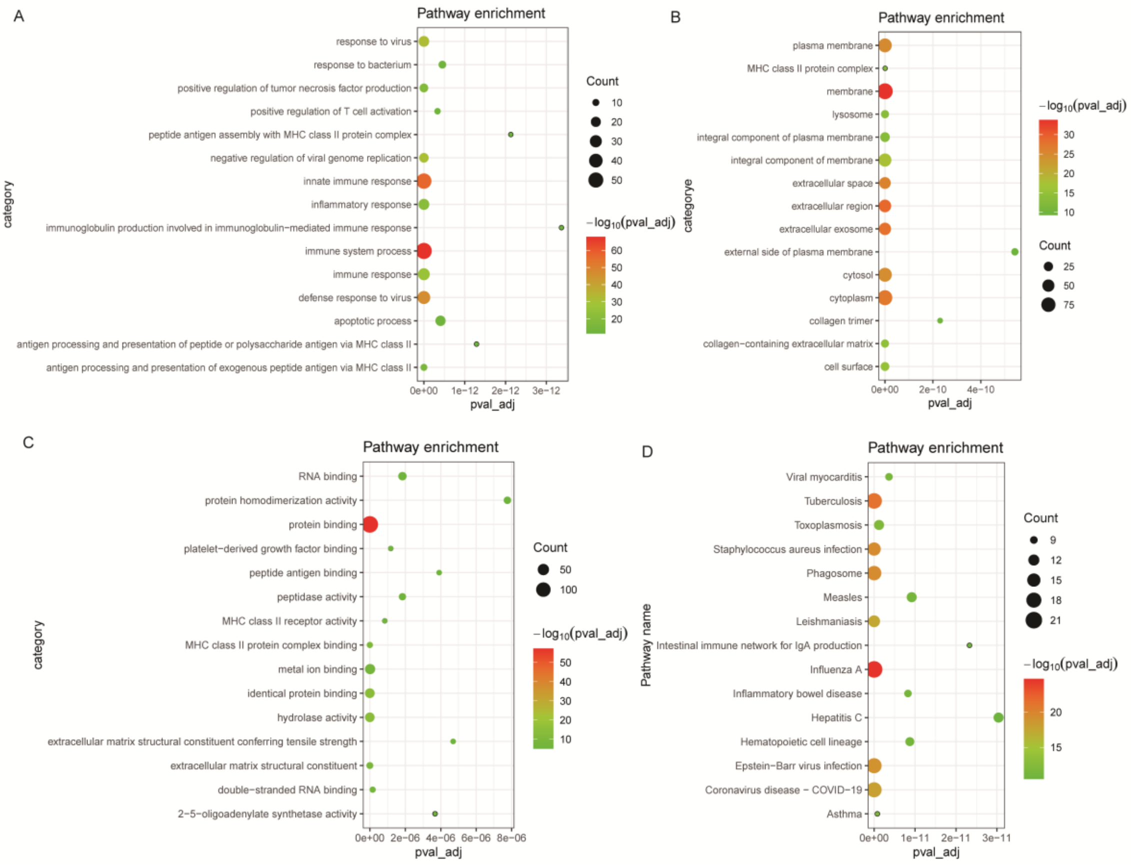

Figure S3: Significantly enriched GO terms and KEGG pathways of DEmRNAs in GSE32591_glomeruli dataset.

A: Top 15 biological process (BP) terms in GO functional enrichment; B: Top 15 cellular component (CC) terms in GO functional enrichment; C: Top 15 molecular function (MF) terms in GO functional enrichment; D: Top 15 significantly enriched pathways in KEGG functional enrichment analysis. The x-axis shows P-value_adj of DEmRNAs enriched in GO terms or KEGG pathways and the y-axis shows GO terms or KEGG pathways. The color scale represented -lg p-value_adj.

Information Classification: General

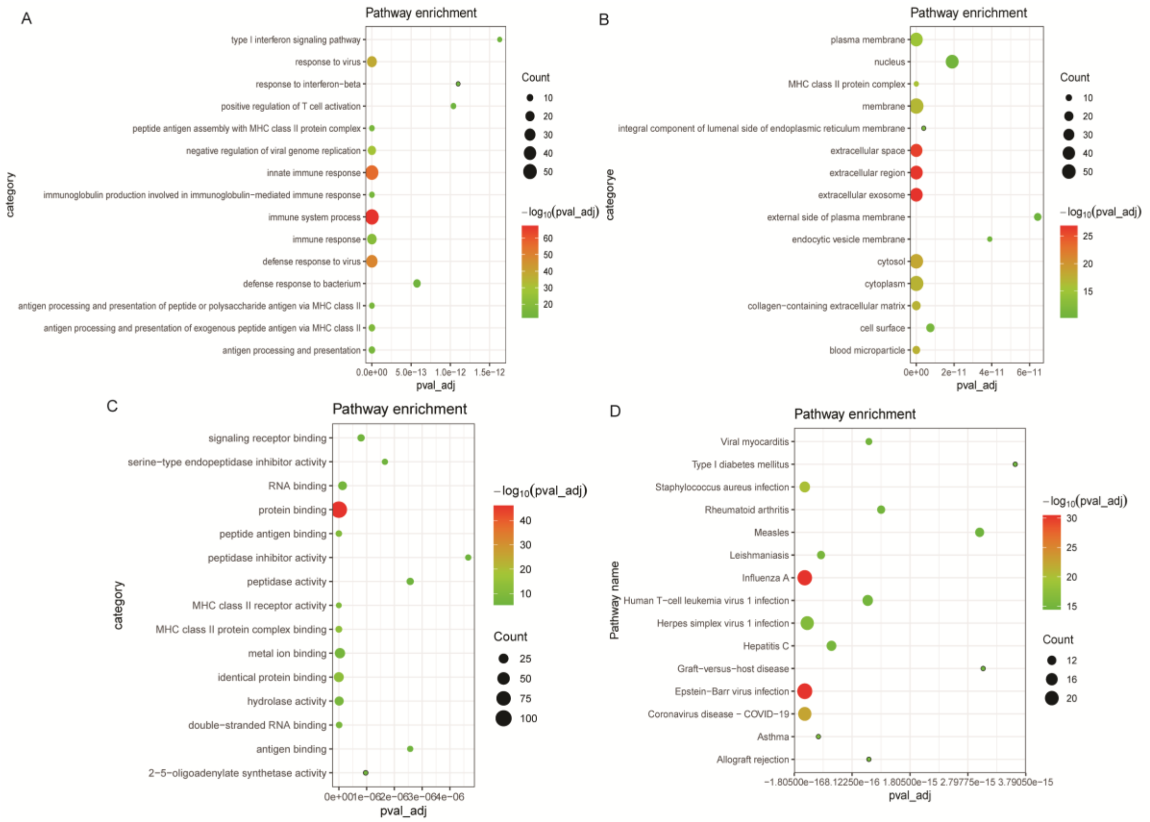

Figure S4: Significantly enriched GO terms and KEGG pathways of DEmRNAs in GSE32591_tubulointerstitium dataset.

A: Top 15 biological process (BP) terms in GO functional enrichment; B: Top 15 cellular component (CC) terms in GO functional enrichment; C: Top 15 molecular function (MF) terms in GO functional enrichment; D: Top 15 significantly enriched pathways in KEGG functional enrichment analysis. The x-axis shows P-value_adj of DEmRNAs enriched in GO terms or KEGG pathways and the y-axis shows GO terms or KEGG pathways. The color scale represented -lg p-value_adj.

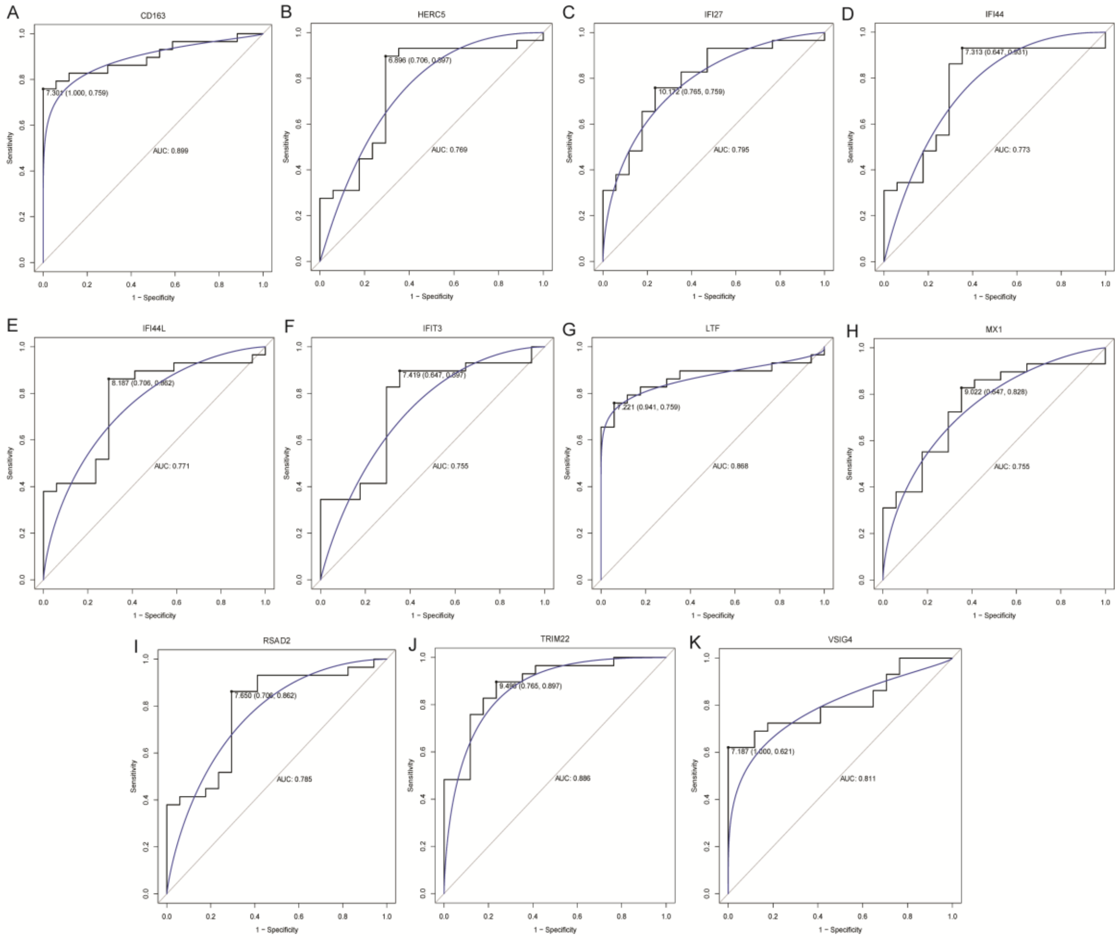

Figure S5: Receiver operating characteristic (ROC) diagnostic analysis of common DEmRNAs in GSE99967_blood dataset.

A: ROC analysis of CD163; B: ROC analysis of HERC5; C: ROC analysis of IFI27; D: ROC analysis of IFI44; E: ROC analysis of IFI44L; F: ROC analysis of IFIT3; G: ROC analysis of LTF; H: ROC analysis of MX1; I: ROC analysis of RSAD2; J: ROC analysis of TRIM22; K: ROC analysis of VSIG4. AUC, area under curve

Information Classification: General

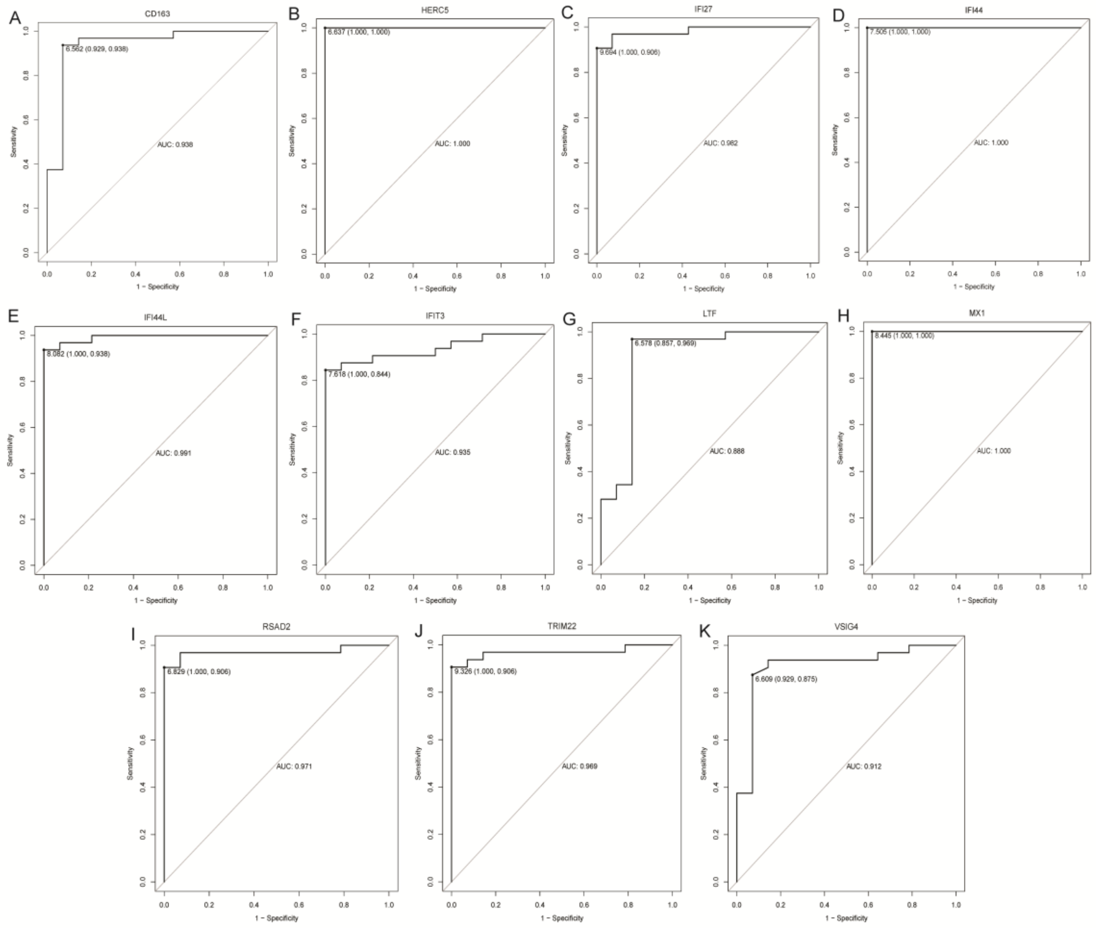

Figure S6: Receiver operating characteristic (ROC) diagnostic analysis of common DEmRNAs in GSE32591_glomeruli dataset.

A: ROC analysis of CD163; B: ROC analysis of HERC5; C: ROC analysis of IFI27; D: ROC analysis of IFI44; E: ROC analysis of IFI44L; F: ROC analysis of IFIT3; G: ROC analysis of LTF; H: ROC analysis of MX1; I: ROC analysis of RSAD2; J: ROC analysis of TRIM22; K: ROC analysis of VSIG4. AUC, area under curve

Information Classification: General

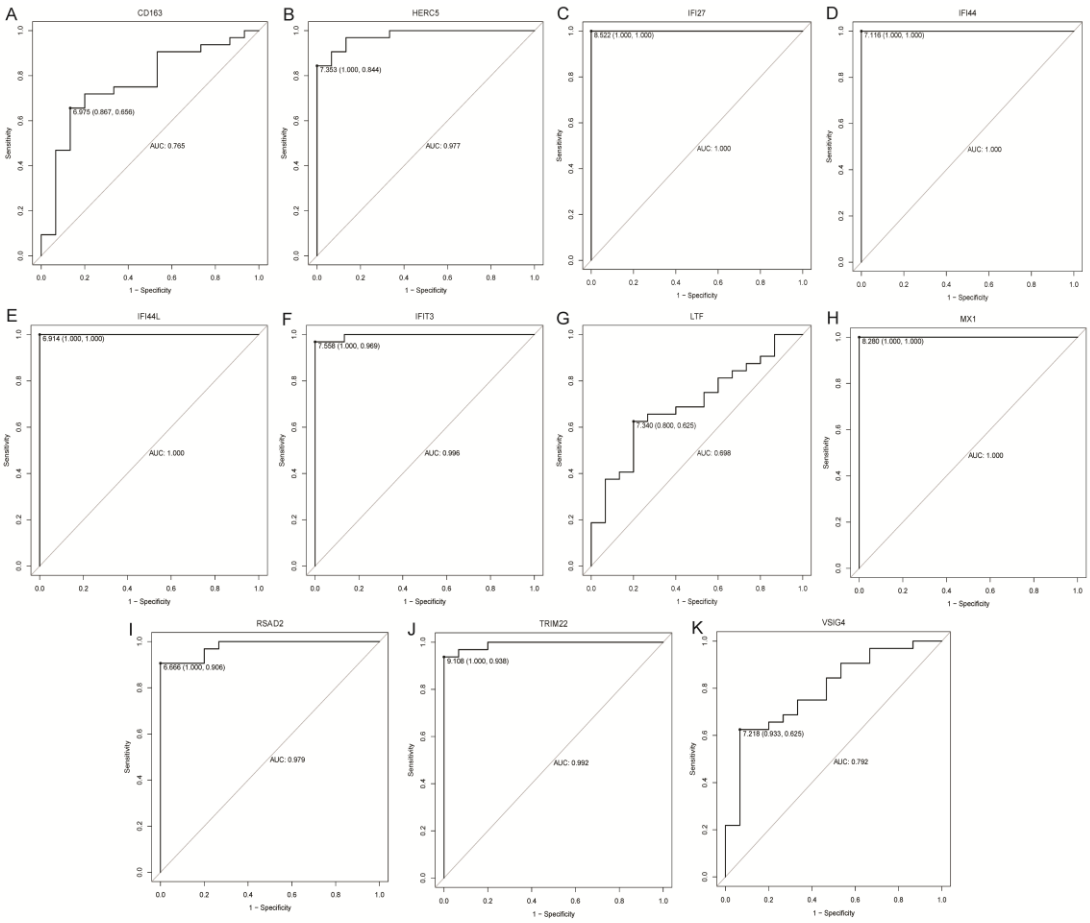

Figure S7: Receiver operating characteristic (ROC) diagnostic analysis of common DEmRNAs in GSE32591_tubulointerstitium dataset.

A: ROC analysis of CD163; B: ROC analysis of HERC5; C: ROC analysis of IFI27; D: ROC analysis of IFI44; E: ROC analysis of IFI44L; F: ROC analysis of IFIT3; G: ROC analysis of LTF; H: ROC analysis of MX1; I: ROC analysis of RSAD2; J: ROC analysis of TRIM22; K: ROC analysis of VSIG4. AUC, area under curve

Information Classification: General

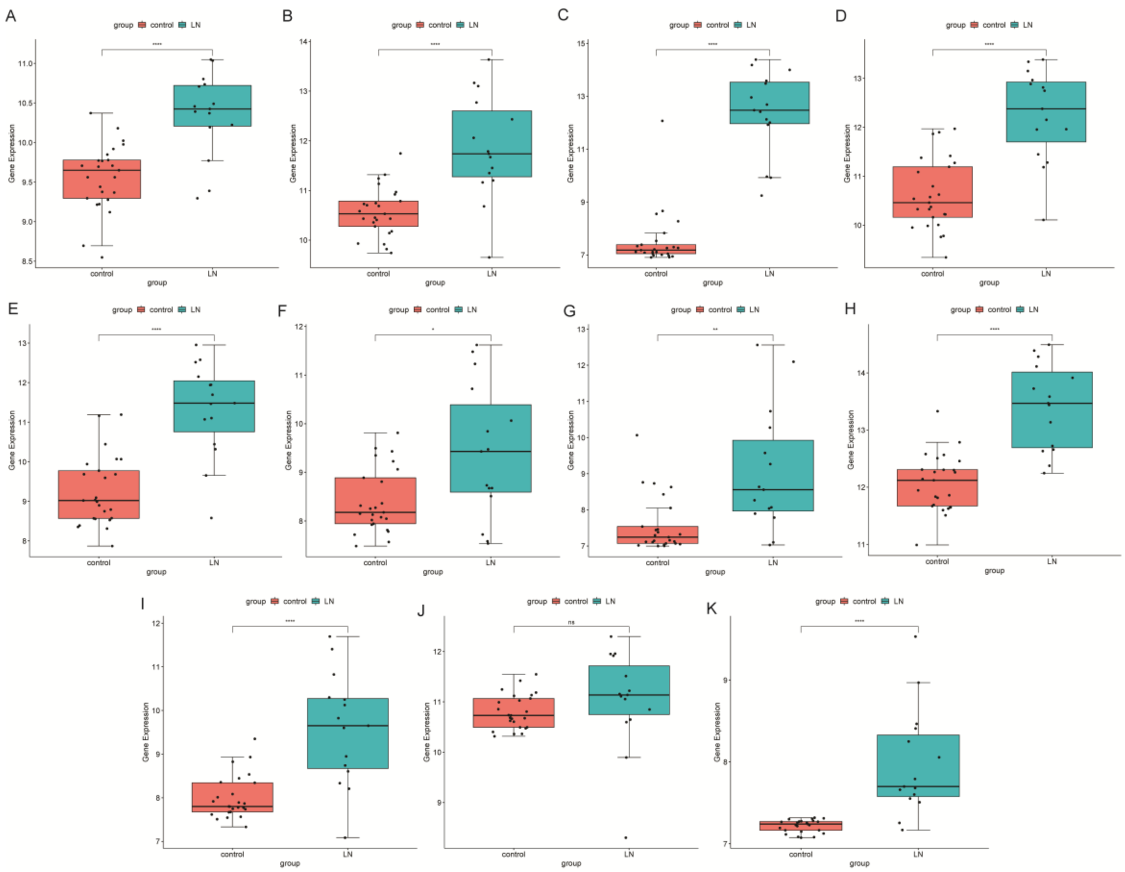

Figure 8: Differential expression of common DEmRNAs in the GSE81622 dataset between the control and the LN groups.

A: Box plot of CD163 expression; B: Box plot of HERC5 expression; C: Box plot of IFI27 expression; D: Box plot of IFI44 expression; E: Box plot of IFI44L expression; F: Box plot of IFIT3 expression; G: Box plot of LTF expression; H: Box plot of MX1 expression; I: Box plot of RSAD2 expression; J: Box plot of TRIM22 expression; K: Box plot of VSIG4 expression. *, $P < 0.05$; **, $P < 0.01$; ****, $P < 0.0001$; ns represents no statistical significance.

Information Classification: General

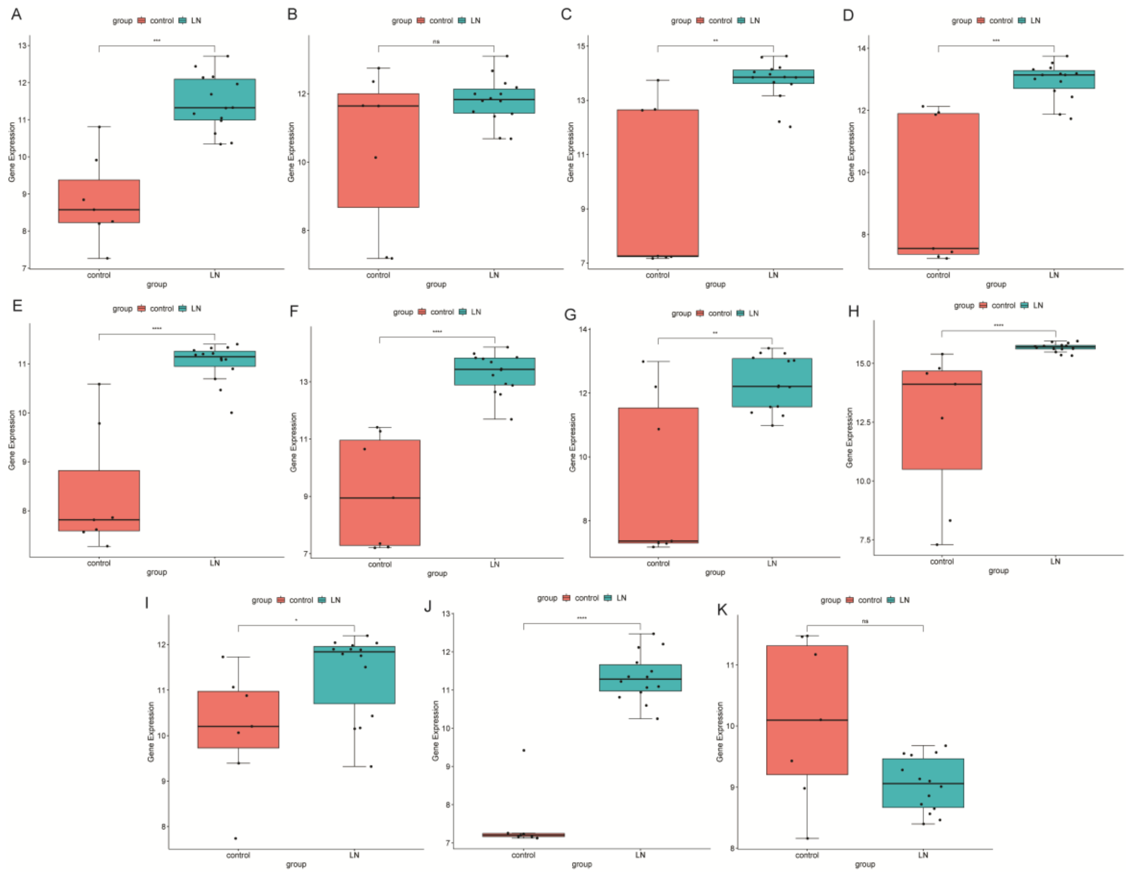

Figure 9: Differential expression of common DEmRNAs in the GSE112943 dataset between the control and the LN groups.

A: Box plot of CD163 expression; B: Box plot of HERC5 expression; C: Box plot of IFI27 expression; D: Box plot of IFI44 expression; E: Box plot of IFI44L expression; F: Box plot of IFIT3 expression; G: Box plot of LTF expression; H: Box plot of MX1 expression; I: Box plot of RSAD2 expression; J: Box plot of TRIM22 expression; K: Box plot of VSIG4 expression. *, P <0.05; **, P <0.01; ***, P <0.001; ****, P <0.0001; ns represents no statistical significance.

Information Classification: General

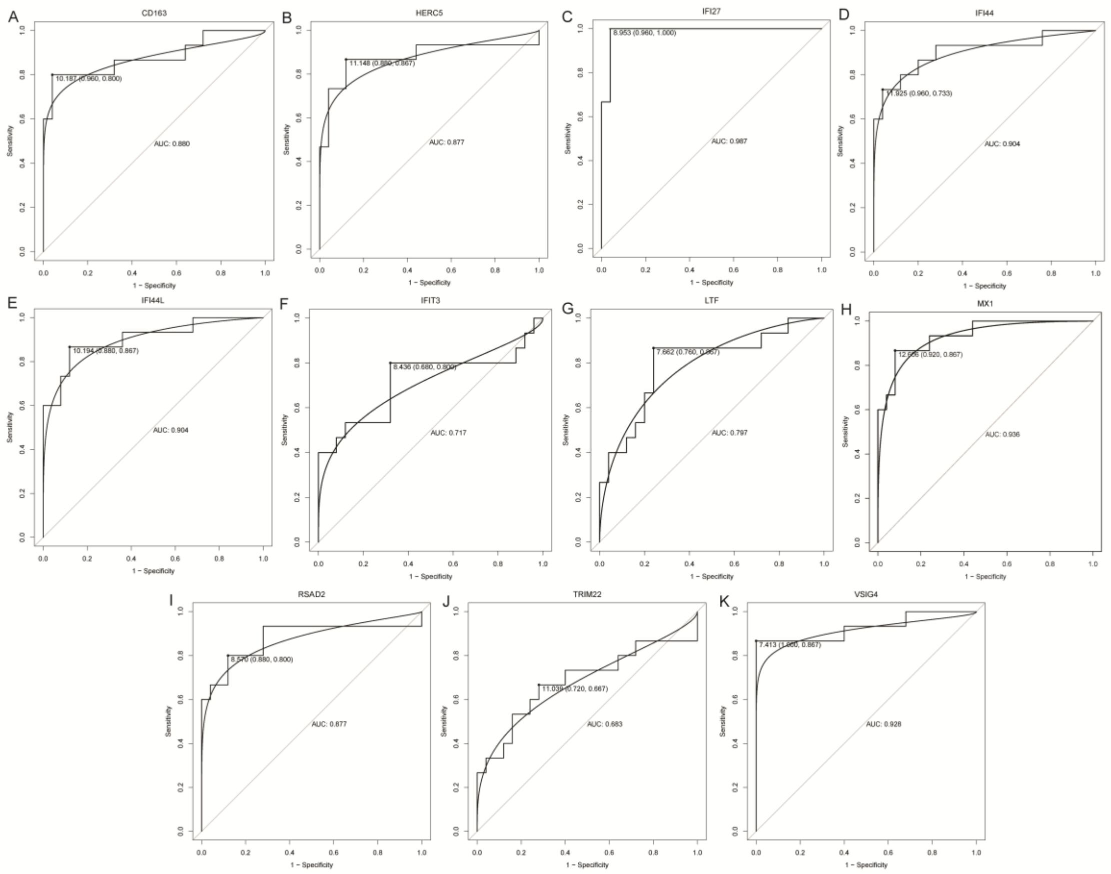

Figure 10: Receiver operating characteristic (ROC) diagnostic analysis of common DEmRNAs in GSE81622 dataset.

A: ROC analysis of CD163; B: ROC analysis of HERC5; C: ROC analysis of IFI27; D: ROC analysis of IFI44; E: ROC analysis of IFI44L; F: ROC analysis of IFIT3; G: ROC analysis of LTF; H: ROC analysis of MX1; I: ROC analysis of RSAD2; J: ROC analysis of TRIM22; K: ROC analysis of VSIG4. AUC, area under curve

Information Classification: General

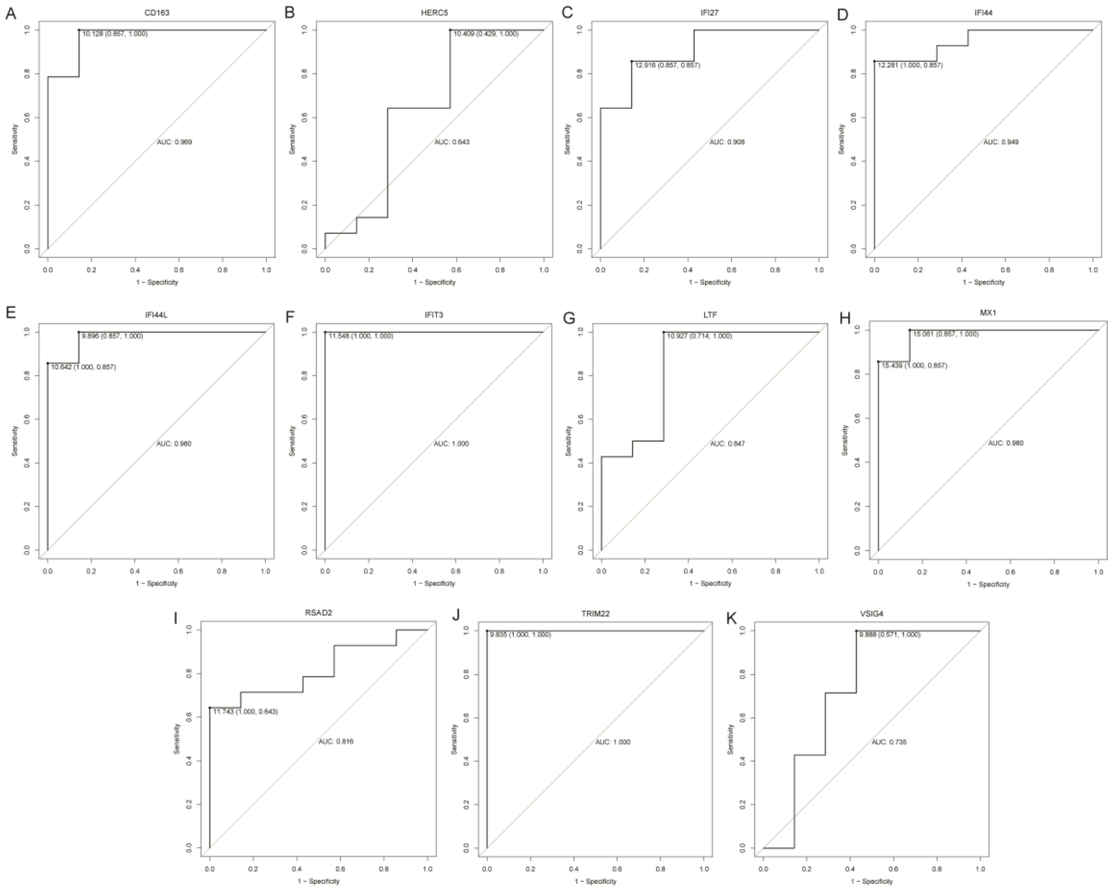

Figure 11: Receiver operating characteristic (ROC) diagnostic analysis of common DEmRNAs in GSE112943 dataset.

A: ROC analysis of CD163; B: ROC analysis of HERC5; C: ROC analysis of IFI27; D: ROC analysis of IFI44; E: ROC analysis of IFI44L; F: ROC analysis of IFIT3; G: ROC analysis of LTF; H: ROC analysis of MX1; I: ROC analysis of RSAD2; J: ROC analysis of TRIM22; K: ROC analysis of VSIG4. AUC, area under curve

Supplementary Table

Table S1: Clinical information of individuals in the RT-PCR

Table S2: GSVA analysis

Table S3: Immune cell types with significant differences in GSE99967_blood, GSE32591_glomeruli and GSE32591_tubulointerstitium datasets

Table S4: Primers used for RT-PCR

Table S5: RT-PCR data

Information Classification: General
